# Stirpiculture

**Published:** 1889-03-16

**Authors:** 


### Stirpiculture.

"Stirpiculture, or the Scientific Propagation of the Human Race," is the title of a pamphlet published a short time back by Victoria Claflin Woodhull, now Mrs. John Biddulph Martin. The authoress complains that people devote their lives to perfecting orchids or cattle, yet will not waste a moment over the solution of perfecting so miserable a creature as man. Although there are institutions to incarcerate the insane, the idiots, the epileptics, the drunkards, and the criminals, there is nowhere a building erected for the purpose of teaching how to perfect the human body. The authoress further asserts that nearly all the crime committed is the result of inherited weak blood, or of inherited malformation, or disease of the brain; and she advises Government to institute an addition to the laws, "Thou shalt not marry when malformed or diseased!" Now heredity has doubtless much influence on both the mental and physical condition of individuals, and it is well known that numerous maladies tend to recurrence; or, using the common expression, "to run in families." Even acquired bad habits may become hereditary, and so be transmitted. But on the other hand, good qualities, both mental and physical, are subject to a similar law. Galton holds that the heredity of any rare and valuable quality from one parent is checked by some opposing quality in the other, and *vice versa*. It is constantly found that the character of skull and other structure, belonging to remote times crops out from some of our numerous ancestors. Instances have been known of persons whose ancestors were dark-skinned, producing dark-skinned children, when they themselves were of the light Saxon type. And so it is with disease. One generation may escape, but after generations may suffer. The authoress's idea that Government should legislate in order to prevent diseased people marrying is somewhat utopian. Even if it were possible to institute such laws against persons evidently and openly diseased, it would not be practical to enforce them against persons latently and inwardly diseased. Yet the latter are as likely to transmit disease as the former. Education and "bringing up" have probably a more powerful influence on after life than heredity. But the term "bringing up" must include suitable mental, moral, and physical regime. In this respect we fully agree with the authoress that very much has not been done, even in the most civilised countries, which should have been done.

Mrs. Victoria Martin asks if it is possible that this temple (meaning the human frame) "has been so defaced and brutalised that one can no longer apply to it the word human? Can it be possible that the masterpiece of God is drugged in opium hells? Are those God's creatures that fill the hot-beds of infamy which swarm in all large towns? Are all those brutish faces that crowd the prisoner's dock a part of Him?" Such queries may be answered according to the lights and beliefs of the reader, and doubtless many different replies will be given. Not so, however, we imagine, with the panacea proposed by the authoress, who thinks women are to put the world into joint again. Mankind, she says, has forgotten the lesson taught by the Hindus five thousand years ago, that to degrade or oppress women involves the physical and moral degradation of man, that the assumption of superiority and of tyranny of the master has "almost extinguished that divine spark in her which alone has the power to regenerate humanity." How this divine spark is to be again fanned into a blaze, and how then the regeneration of humanity is to be effected by female power, is not very clearly explained in the pamphlet under notice. We fancy the problem of making the world good has not been completely solved by Mrs. Victoria Martin.

